# A multi-center longitudinal study on responsive breastfeeding in China from the perspective of health equity: research protocol

**DOI:** 10.1186/s12939-021-01430-5

**Published:** 2021-05-01

**Authors:** Wen Shu, Menglong Li, Nubiya Amaerjiang, Xin Fan, Shunna Lin, Sofia Segura-Pérez, Rafael Pérez-Escamilla, Yifei Hu

**Affiliations:** 1 Department of Child, Adolescent Health and Maternal Care, School of Public Health, Capital Medical University, No.10 You’anmenwai Xitoutiao, Fengtai District, Beijing, 100069 China; 2 Department of Child Healthcare, Chongqing Health Center for Women and Children, Chongqing, 401147 China; 3 Department of Pediatrics, Tianhe District Maternal and Child Hospital of Guangzhou, Guangzhou, 510620 China; 4 Nutrition Unit, Hispanic Health Council, 175 Main St., Hartford, CT 06106 USA; 5 Yale School of Public Health, Yale University, New Haven, CT 06510-3201 USA

**Keywords:** Breastfeeding, Infant growth and development, Infant behavior and cognition, Parenting stress, Health equity, Responsive breastfeeding

## Abstract

**Background:**

Socio-economic inequities can strongly influence suboptimal infant feeding outcomes. Factors such as lack of knowledge about breastfeeding, low family income, low educational attainment, social and economic status, cultural norms and ethnicity may negatively affect success with offering breastfeeding following a responsive feeding approach (ie. responsive breastfeeding). Such inequities can indeed shorten breastfeeding duration, and negatively affect behavioral and cognitive infant outcomes. In China, there is a dearth of studies focusing on breastfeeding from the responsive and health equity perspective.

**Objective:**

The aim of this article is to present a protocol of an ongoing longitudinal cohort study investigating factors associated with responsive breastfeeding behaviors, and the child’s behavioral and cognitive development from birth to12 months post-partum in five centers in China. The study seeks to identify breastfeeding barriers and facilitators from a health equity perspective.

**Methods:**

We are enrolling 700 women and their singleton full term infants in Chongqing, Huizhou and Guangzhou urban and rural areas. The study questionnaires will be administrated within 72 h, 30 days, 3, 6, 9, and 12 months post-partum during the baby’s vaccination visits. We will investigate the difference between urban and rural areas sociodemographic characteristics, breastfeeding knowledge, attitudes and practice, postnatal depression, maternal emotion regulation and parenting stress, and anthropometric and cognitive development indicators of the infants at each time-point.

**Conclusion:**

Our article illustrates how a cohort study can be designed to understand the barriers and facilitators of responsive breastfeeding taking equity principles into account to help promote infants’ growth and development in China.

**Table:** 

This article is a part of the Interventions and policy approaches to promote equity in breastfeeding collection, guest-edited by Rafael Pérez-Escamilla, PhD and Mireya Vilar-Compte, PhD	

## Background

In the past ten years, health equity has been emphasized more in the maternal-child care research agenda. Health equity underlies a commitment to reduce—and, ultimately, eliminate—disparities in health and in its determinants, especially the social determinants [1]. Pursuing health equity means striving for the highest possible standard of health for all people and giving special attention to the needs of those at greatest risk of poor health, based on social conditions [2]. This approach is crucial for human development as child malnutrition and poor health are intimately linked with each other and with suboptimal child growth and development [3].

Breastfeeding is beneficial to the health of mothers as well as their infants, including their overall development [4]. Indeed, breastfeeding can prevent infections and malocclusions, improve infants’ intelligence, and may reduce the risk of overweight and diabetes among children [5]. In addition, protecting, promoting and supporting breastfeeding is critical to achieving the sustainable development goals (SDG) in low- and middle-income countries by 2030 [6], including the tenth goal of SDG, which is to reduce inequality [5]. Previous studies have evaluated the barriers to breastfeeding [7] and the effectiveness of strategies to promote it [8, 9]. However, there is a dearth of studies designed on how to support breastfeeding and other aspects of responsive feeding from the health equity perspective in China.

Protecting, promoting and supporting breastfeeding requires overcoming major health care and social protection systems challenges worldwide. In China, a wide range of social determinants of health, as well as psychological and geographical factors have been identified as barriers for exclusive breastfeeding [7, 10–12]. A cross-sectional study conducted in China identified as key factors for breastfeeding: breastfeeding education, family support, health care institutional policies and practices that are conducive to breastfeeding, paid maternity leave and other maternity protection benefits [13]. Indeed, a quasi-experiment study in China found that increasing breastfeeding rate requires family-centered breastfeeding education and support [10]. Another study reported that most mothers encountered breastfeeding barriers that might interrupt exclusive breastfeeding when they left the health facility [14] and that pre- and post-natal breastfeeding education and supporting form healthcare providers are needed to facilitate and sustain breastfeeding.

Mother’s psycho-emotional status also affects breastfeeding outcomes. Postpartum stress has been shown to affect infant growth, nutrition, bonding, temperament and ultimately mental wellbeing during childhood [15]. The association between mother’s psychological health and infants’ cognitive behavior is noteworthy. For example, higher maternal paranoid ideation may delay language and cognitive development. Furthermore, maternal hostility and anxiety increase gross motor developmental delays, and maternal depression leads to overall development delays in infants [16].

Responsive feeding (RF), defined as “feeding practices that encourage the child to eat autonomously and in response to physiological and developmental needs, which may encourage self-regulation in eating and support cognitive, emotional, and social development” [17], is an aspect of responsive parenting that contributes to a nurturing and caring environment for children [18]. RF principles can guide parents on how to feed their young children, which complements existing global guidance on what to feed them [17, 18]. Parents who practice RF identify and respond to their infants’ hunger and satiety cues in a nurturing manner, fostering healthy eating habits and self-regulation, and reducing excessive weight gain risk [19]. The nurturing environment created by RF has immense potential for improving child growth and development outcomes [20], especially in settings such as China where poverty and malnutrition in all its forms (including stunting, overweight/obesity, and micronutrient deficiencies) coexist [21].

The 2016 Lancet Early Childhood Development Series highlighted that a lack of nurturing care in early childhood, including responsive parenting and feeding, can have lifetime health, developmental, and productivity implications [22]. There is a paucity of evidence on how best to design and deliver home-based parenting skills interventions that have been shown to be key for improved child psycho-emotional, social and cognitive development [23], with its benefits extending to adulthood [24].

Breastfeeding research in the context of RF has become a priority given the vital role that breastfeeding plays in infants’ growth and development, including cognition [25]. Even though the relationship between breastfeeding and improved cognition has been documented for decades across different countries [25–27], few countries have operationalized breastfeeding promotion programs in the context of nurturing care. Research is needed to understand how best to do so as globally breastfeeding services remain fragmented and of relatively low quality. Furthermore, programs at scale are rare and poorly evaluated.

### Aims and objectives

At present, there is still a dearth of research to explore simultaneously factors influencing breastfeeding and early childhood development. This work is needed to understand how best to integrate maternal-child health, nutrition and development programs. We are unaware of studies that have been conducted in China to address this knowledge gap. In order to fill this research gap, we designed a multi-center longitudinal cohort study from birth to 12 months of age taking health equity principles into consideration with the goals to: a) to understand RF barriers and facilitators including those related to breastfeeding, and b) determine the association between breastfeeding and infant development in China. The study’s research hypotheses are that in Chinese households: 1) there are barriers to responsive feeding, driven by the social determinants of health, 2) suboptimal responsive feeding can shorten the duration of breastfeeding and delay infant’s growth and development.

## Methods/ design

### Study design: multi-center prospective longitudinal study

We will follow up women who delivered a singleton full term infant of age at 72 h, 30 days, 3 months, 6 months, 9 months and 12 months after birth, with these time points corresponding to each of the six study waves. At each point we will collect information on maternal knowledge, attitude and practice (KAP) towards breastfeeding, postnatal depression, emotional regulation and parenting stress. We will use questionnaires responded by the mothers to assess their psychological status and emotional regulations, and we will extract data from the infants’ medical charts to assess their growth and cognitive development. The recruitment of participants began in July 2019 and is currently on hold due to the coronavirus disease 2019 (COVID-19) pandemic. This study was registered at the Chinese Clinical Trial Registry (ChiCTR) (record ID; http://www.chictr.org.cn (ChiCTR2100044028)).

### Study setting

**Fig. 1:**
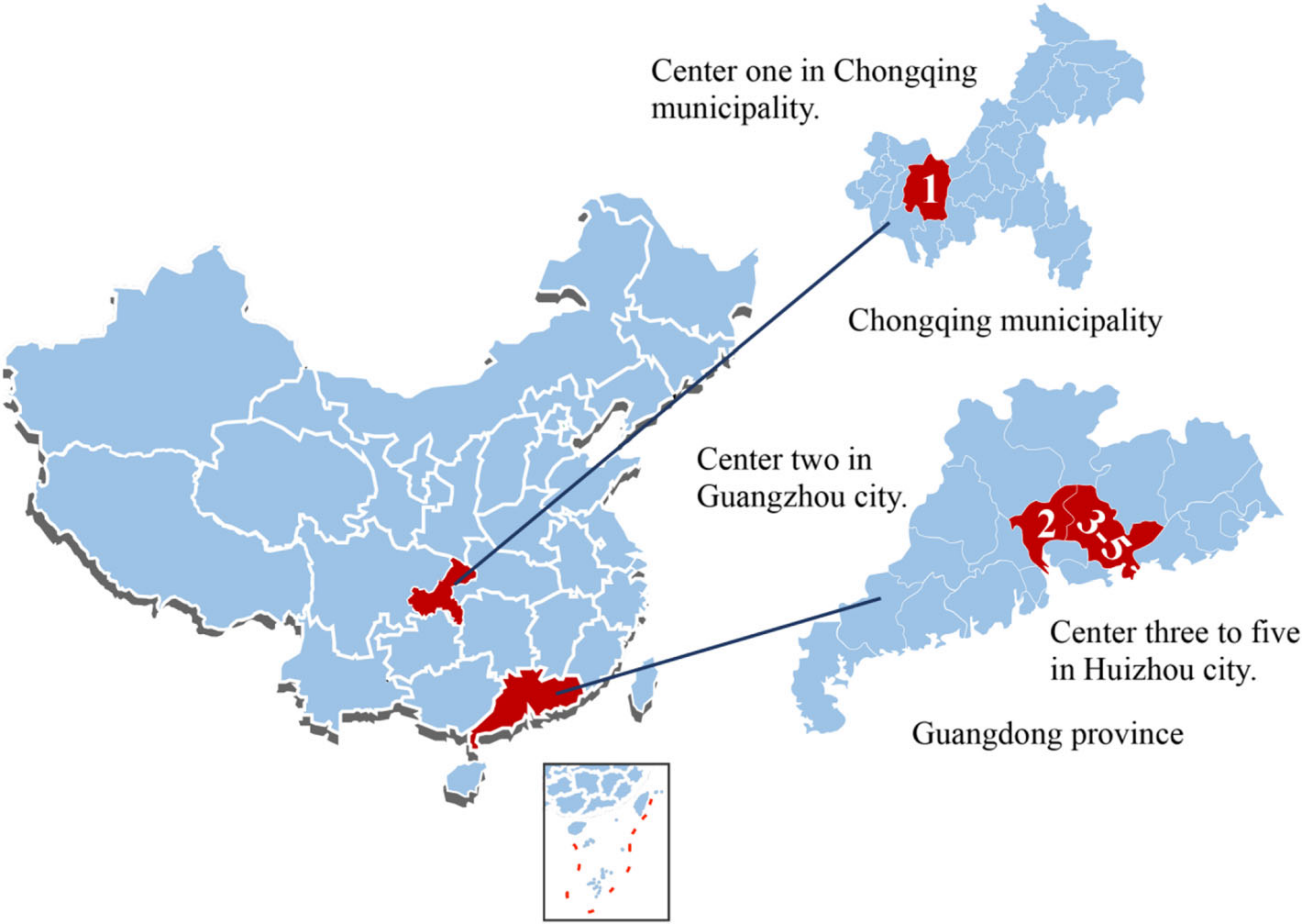
The multi-center’s location for the longitudinal study on responsive feeding in China

We have started recruiting participants from five public health institutions in Chongqing municipality, Guangzhou city and Huizhou city of Guangdong province representing Southern China. Public hospitals in China have more economic resources and advanced technology than private facilities, hence the public health care system is able to provide more comprehensive and effective medical services than the private health care system. Center one, Chongqing Health Center for Women and Children is located in Yubei district, Chongqing, western China, it serves over 10,000 infants in the regions annually. Center two, Tianhe District Maternal and Child Hospital of Guangzhou is located in Tianhe district, Guangzhou, that serves 1500–2000 deliveries annually. Centers three to five are primary health facilities that jointly represent Huizhou city in our study, namely Huidong County Maternal and Child Health Service Center, Tieyong Town Health Center and Duozhu Town Health Center. Centers one and two targets urban population and the third center serves a mixed urban and rural population. Centers four and five targeting rural populations, located in Huidong county. The diversity of populations represented on our study sample that includes western and eastern of China and both rural and urban areas will facilitate our aim to understand breastfeeding risk factors from the health equity perspective (Fig. 1).

The principal institution responsible for the study is the Department of Child, Adolescent Health and Maternal Care, School of Public Health, Capital Medical University located in Beijing.

### Participants and selection criteria

At present, China’s government offers children free and compulsory vaccinations, including the vaccines for Hepatitis B, Bacillus Calmette-Guerin, Diphtheria, Pertussis and Tetanus that are delivered in primary public medical institutions during the first year of life. We plan to recruit 700 women delivering singleton infants who are willing to attend five research centers during regular vaccination days and willing to participate in six rounds of follow-up surveys. We will use each contact opportunity to remind doctors and parents about their next follow-up survey appointment. Inclusion criteria are women: (1) aged 18–50 years with full-term singleton birth; (2) living in the target neighborhood for at least 1 year, (3) can continue visit one of the five study centers to receive healthcare services at least until 12 months after birth. Exclusion criteria are: (1) mothers that delivered twins or multiple birth; (2) premature birth; (3) babies with congenital diseases; (4) maternal mental disorders or difficulty understanding and completing the study questionnaires; (5) known diseases that could affect breastfeeding.

### Sample size calculation

From the perspective of health equity, we designed the study to enroll participants from rural and urban areas. We estimate the sudy sample size based on the different prevalences of breastfeeding in urban an rural areas.

The sample size was calculated using the following formula for comparison of two proportions:

$$ {\mathrm{n}}_{\mathrm{urban}}={\mathrm{n}}_{\mathrm{rural}}=\frac{{\left[{\mathrm{Z}}_{1-\upalpha /2}\sqrt{2\overline{\mathrm{p}}\left(1-\overline{\mathrm{p}}\right)}+{\mathrm{Z}}_{\upbeta}\sqrt{{\mathrm{p}}_1\left(1-{\mathrm{p}}_1\right)+{\mathrm{p}}_2\left(1-{\mathrm{p}}_2\right)}\right]}^2}{{\left({\mathrm{p}}_1-{\mathrm{p}}_2\right)}^2} $$

where *n*_urban_ and *n*_rural_ are the sample sizes corresponding to the urban and rural groups, respectively; *Z*_*1-α/2*_ and *Z*_*β*_ are the Z values corresponding to the alpha (*α*) and beta (*β*) error, respectively; *p*_1_ and *p*_2_ are the estimated proportion of breastfeeding rates in urban and rural areas, respectively, while $\overline{p}$ is the average of *p*_1_ and *p*_2_.

According to the China Development Research Foundation (CDRF) 2019 *Report on National Survey of Factors Influencing Breastfeeding*, the prevalence of breastfeeding during the first six months of life in large cities (estimated *p*_1_) and medium/small cities (estimated *p*_2_) was 36 and 23%, respectively. With a statistical power of 90% and a two-tailed significance level of 5%, we estimated that a minimum of 257 participants for each group (urban and rural), should be recruited respectively. Therefore, a sample of 514 are required to observe a 13% difference in the prevalence of breastfeeding between the two groups. Taking into consideration that 25% participants may be lost to follow-up, our goal is to enroll 700 participants.

### Recruitment procedures

Centers’ obstetricians, midwives and/ or experienced nurses will review and screen the information of the women who have delivered within the previous 72 h following the study’s enrollment criteria. The study will then be explained to those mothers who meet the criteria with the help of a leaflet that briefly and clearly explains the study. Enrolled participants will swipe a quick response code (QR code) to provide their web-based informed consent and register into the survey system using the WeChat App. The informed consent form explains in detail the study procedures and includes statements on the benefits and harms regarding study participation. It specifically presents information on potential risk, and confidentiality and privacy protection measures. All participants will be reassured that their participation is voluntary and that they are free to withdraw at any wave of the study without giving any reason, and that their decision to participate or not will not affect the clinical care for them or their infants. The administration of each survey was designed to not exceed 30 min. Before the survey started, all staff received a one-hour on-line training that included a formal assessment to confirm that staff had a full understanding of the project research methods, confidential management of information, and quality control system. The protocol for this study was approved by the Ethics Committee of Capital Medical University (No. Z2019SY001).

### Data collection

**Fig. 2:**
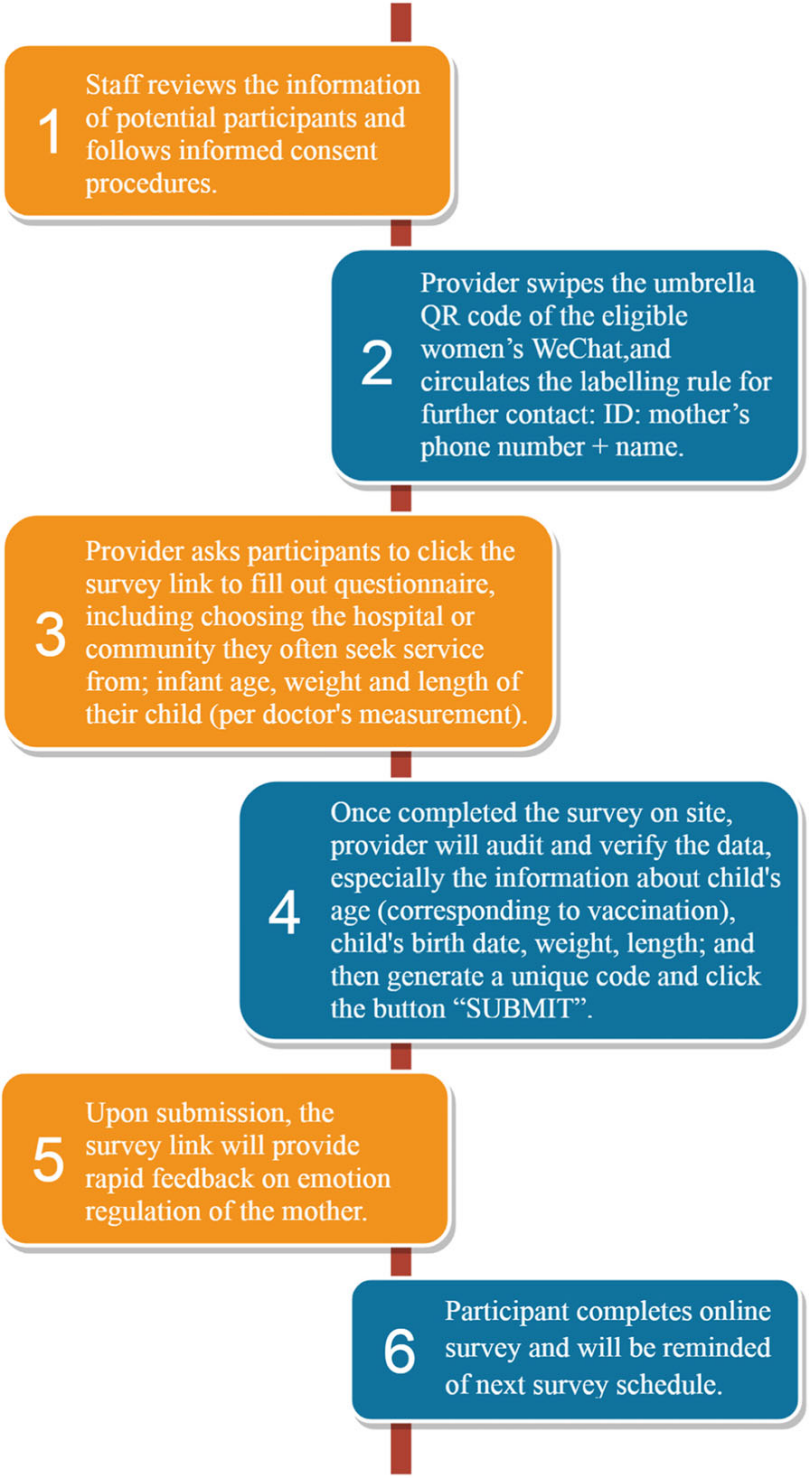
The 6-wave survey procedure of data collection for the multi-center longitudinal study on responsive feeding in China

The electronic self-administered questionnaires were developed by team members with strong expertise in maternal-child public health nutrition. All data will be collected via an online questionnaire management system (www.wjx.cn) popular and widely used in China, and that is similar to SurveyMonkey®. We developed the electronic version of all the self-administered questionnaires before recruitment started, pretested and released them for participants to fill in on the study’s secure web-based system. Local doctors who are responsible for recruitment will undertake quality control for the survey and will ensure only the eligible participant could access to the survey system. This approach has been successfully implemented in other studies with similar populations in China [28–30]. The specific procedures followed across the six survey waves are shown in Fig. 2. The first survey (wave 1) will be completed within 72 h after delivery on-site under the supervision of a health care provider. Participants can fill out the questionnaires for the remaining five surveys either on-site or at home by clicking the web link forwarded to them.

The timeline and contents of the questionnaire are summarized per study wave in Table 1. Data collected will includes mother’s KAP related to breastfeeding, postnatal depression, emotional regulation and parenting stress; as well as infant’s growth (weight, length) and development status, including cognition. Data on barriers for breastfeeding and RF in general will include self-reported healthcare services access and socioeconomic status.

**Table 1:** Timeline of questionnaire application by study wave in the multi-center longitudinal study on responsive breastfeeding in China

Online questionnaire content	Wave 1 (1st survey)	Wave 2 (2nd survey)	Wave 3 (3rd survey)	Wave 4 (4th survey)	Wave 5 (5th survey)	Wave 6 (6th survey)
	within 72 h pp	1 month pp	3 months pp	6 months pp	9 months pp	12 months pp
Informed consent	Mothers	Mothers	Mothers	Mothers	Mothers	Mothers
Socio-demographic information	Both	Both	Both	Both	Both	Both
Knowledge, attitude and practice towards breastfeeding	Mothers	Mothers	Mothers	Mothers	Mothers	Mothers
Infant feeding style questionnaire (IFSQ) [31]	Mothers	Mothers	Both	Both	Both	Both
Edinburgh postnatal depression scale (EPDS) [32]	Mothers	Mothers	–	–	–	–
Emotion regulation questionnaire (ERQ) [33]	Mothers	Mothers	Mothers	Mothers	Mothers	Mothers
Parenting stress index-short form (PSI-SF) [34]	–	–	–	Mothers	Mothers	Mothers
Caregiver reported early childhood development instruments (CREDI) [35], reported by mothers	–	–	Infants	Infants	Infants	Infants

Note: The information of mothers and infants will be collected simultaneously; the content of each questionnaire is detailed in its corresponding area of the methods/ design section. All the scales that used have been translated and pre-tested in Chinese

Abbreviation: pp., postpartum

### Measurement of outcomes, independent variables and covariates

The outcome indicators are based on growth and developmental indicators such as infants’ weight, length, and child development score based on the Caregiver Reported Early Childhood Development Instruments (CREDI) [35].

The key independent variables are the following mother’s RF-related behaviors: mother’s KAP towards breastfeeding including exclusive breastfeeding or not; duration of breastfeeding; pressuring child to eat, restricting what child eats, and responsive styles according to infant feeding style questionnaire (IFSQ) [31]; postnatal depression scores; emotion regulation scores; parenting stress scores.

The co-variates included are based on household socio-demographic data including socioeconomic status and household geographical location and corresponding health center.

### Socio-demographic data of mothers

Socio-demographics will be collected from wave 1 to wave 6 via the self-administered questionnaires. The information collected in wave 1 will include maternal date of birth, marital status, maternal educational attainment, current employment status, duration of maternity leave, household monthly income, height and weight at beginning of pregnancy, weight gain during pregnancy, pregnancy complications and smoking status during pregnancy. The data on current employment and maternity leave status will be also be collected at waves 2, 3 and 4. The information on date of birth and current employment status will be collected for verification purposes at waves 5 and 6.

### Knowledge, attitude and practice towards breastfeeding of mothers

The data on KAP towards breastfeeding assessed in wave 1 will include source of breastfeeding knowledge, infant feeding patterns, colostrum feeding, formula feeding, exclusive breastfeeding, frequency of breastfeeding or formula feeding. Data will also be collected on difficulties encountered during breastfeeding and if any, to whom the mother turns to for support, intentions with regards to breastfeeding or formula feeding, introduction or feeding of solids, other beverages. Data on infant feeding practice will be collected in waves 2–6. It will include breastfeeding status, frequency of breastfeeding and difficulties encountered during breastfeeding. Mothers no longer breastfeeding will be asked to report when they stopped breastfeeding, frequency, dosage, and duration of formula feeding and other fluids and solids, if any, will also be collected. The reason for introducing and frequency of solid foods consumption will be documented in waves 3–6.

### Feeding beliefs and behaviors measurement: infant feeding style questionnaire (IFSQ)

The IFSQ was designed to measure infant feeding beliefs and behaviors of mothers and infants via estimating latent factors for five feeding styles, namely laissez-faire, restrictive style (RS), pressuring style (PR), responsive style (RP) and indulgent, with 39 questions on maternal beliefs (coded on a 5-point Likert scale: disagree, slightly disagree, neutral, slightly agree, agree) and 44 questions on behaviors (coded on a 5-point Likert scale: never, seldom, half of the time, most of the time, always) [31]. Data for estimating the latent food behavior factors will include in waves 1 and 2: finishing (PR8), cereal (PR12–15) and soothing (PR17,19) for pressuring style; amount (RS4) and diet quality (RS8–11) for restrictive style; and satiety (RP6–7) and attention (RP12) for responsive style. In waves 3–6 data collected will include finishing (PR1–3), cereal (PR11) and soothing (PR16) for pressuring style; amount (RS1–2) and diet quality (5–6) for restrictive style; and satiety (RP1–5) and attention (RP8–11) for responsive style.

### Postnatal depression screening: Edinburgh postnatal depression scale (EPDS)

The EPDS is a 10-item self-reported scale that will be used to screen for postnatal depression [32] in waves 1–2. The mothers in the study will be prompted to choose the response options that best defines the frequency of occurrence over the past 7 days of the different depression symptoms listed. Specifically, they will need to choose either never, occasionally, often and always; and a corresponding value ranging from 0 (never) to 3 (always) will be assigned to each scale item, based on the response option selected. Subsequently, a summative score ranging from 0 to 30 will be computed for each mother. The higher the total scores, the stronger the risk of being depressed. Mothers will be classified as follows: in need for referral if scale scores ≥9; needs more in-depth diagnosis if scale scores ≥13; likely to be in need for psychiatric treatment if the 10th-item (‘the thought of harming myself has occurred to me’) score is ≥1.

### Emotion regulation: emotion regulation questionnaire (ERQ)

The ERQ is designed to assess the capacity for emotion regulation via 10-item statements from the perspective of cognitive reappraisal or expressive suppression [33]. The cognitive reappraisal facet is composed by 6 statements, with the remaining 4 statements focusing on expressive suppression. Each statement is responded with a 7 Likert menu with the item score ranging from 1 to 7; the higher value indicates that the strategy being probed for is being used more frequently emotion regulation. Higher cognitive reappraisal is linked to positive patterns of affect, social functioning, and life satisfaction [36]. By contrast, emotional suppression is associated with negative affect and maladaptive action [37]. The ERQ will be assessed in each wave of the survey to be able to examine the association of maternal emotion regulation with infant feeding practices.

### Parenting stress assessment: parenting stress index-short form (PSI-SF)

The PSI-SF is a brief and well-validated clinical and research 36-item tool to assess parenting stress [34]. The 5-point Likert response options for each item range from “strongly disagree” to “strongly agree” corresponding to scores from 1 to 5; the higher the score, the greater the levels of parenting stress are. Parenting stress of mothers will be conducted in waves 4–6.

### Socio-demographic and biomedical information of infants

The socio-demographic information of the children will be abstracted from the birth certificate files in wave 1; these will include sex, birth weight, recumbent length, birth date, pregnancy duration, mode of delivery. The data on infant growth will be based on the weight and length measurements that will be conducted by pediatricians when infants visit the study center for vaccination in waves 2–6.

### Cognitive assessment of infants: caregiver reported early childhood development instruments (CREDI)

The CREDI was developed by Harvard University to assess the cognitive development of infants and toddlers aged 0–35 months. The scale is divided into 6-months age intervals and includes 20 questions at each interval [35]. The portion for the scale corresponding to infants aged 0–12 months will be included in this study. Part A of the scale targeting 0–5 months old infants will be conducted in wave 3, part B focusing on aged 6–11 months old infants in wave 4–5, and part C for 12–17 months old infants will be conducted in wave 6. This will allow us to generate prospective data on the evolution of the children’s motor, psycho-social and cognitive development. Since there is a lack of breastfeeding studies in China addressing responsive feeding, we had to use internationally accepted scales to study responsive feeding approaches to breastfeeding (i.e. responsive breastfeeding) and other infant feeding practices among Chinese infants. As far as we know, this will be the first time such scales are used in China.

### Data management and quality assurance

The original data will be stored in the questionnaire system under the principal investigator’s (PI) account and can only be accessed and downloaded by authorized team members of this study. In order to facilitate the management of the research centers ability to tracking participants, the sub-accounts of each center will provide authority only to view the data collected but will not allow for any changes or deletion of data. The questionnaires applied across the 6 waves will be linked using the name and phone number of mothers, their research center and the vaccination schedule of the infants. The PI will carry out data quality control measures, and each research center will receive feedback on their data collection efforts every two weeks. The study’s information system will also help the hospitals track the study’s participants to minimize dropouts. The follow-up tracking system will be implemented by local staff based at each of the five research centers. The participants’ contact information have and will continue to be shared with the PI who will then enter it in the study’s database. All study data will be stored in a password protected Excel file with double backup. We have already learned that the management of the participants in the research center needs to be improved as some have missed some waves of the study. The solution is to follow up with them through the online survey system described in data collection section.

### Current progress and optimization

Recruitment in the five research centers was suspended since January 2020 due to the COVID-19 outbreak when most clinical services were halted, and strong social distancing and isolation measures began to be implemented. The study will restart in 2021. So far, a total of 104 participants have enrolled in the study, 92 completed the wave 1 survey and 12 participants completed the other waves of the study but missed the baseline survey due to missing the short 72-h window of the wave 1 survey. The average age of the recruited mothers is 29.3 ± 4.2 years, the average height is 159.8 ± 5.3 cm, and the average pre-pregnancy weight is 54.4 ± 9.5 kg (Table 2).

**Table 2:** Descriptive analysis of the participants in the multi-center longitudinal study on responsive breastfeeding in China as of October 2020 (*N* = 104)

Basic information	mean ± SD / n (%)
Study setting	
Chongqing	57(54.8)
Huizhou	31(29.8)
Guangzhou	16(15.4)
Study wave	
1	92(88.5)
2	26(25.0)
3	16(15.4)
4	7(6.7)
5	0
6	1(1.0)
Age at delivery (y)	29.3 ± 4.2
Pre-pregnancy height (cm)	159.8 ± 5.3
Pre-pregnancy weight (Kg)	54.4 ± 9.5
Marital status (%)	
Yes	90(88.5)
No	2(1.9)
Education (%)	
Primary school or below	0
Middle school	14(13.5)
High school	17(16.3)
University or above	61(58.7)
Monthly income (%)	
≤ 5000	22(21.2)
5001–10,000	42(40.4)
10,001–20,000	22(21.2)
> 20,000	5(4.8)
Unknown	1(1.0)
Current work status (%)	
Working	16(15.4)
Not working	26(25.0)
On maternity leave	50(48.1)
Maternity leave duration	
< 3 months	7(6.7)
4–6 months	48(46.2)
> 7 months	4(3.8)
None	11(10.6)
Works at home	11(10.6)
Not working for 1 year	11(10.6)

### Statistical analysis

Descriptive analysis will be performed to describe the socio-demographic characteristic of the participants and to summarize the scale scores of the questionnaires. In order to identify the inequity factors of RF, the crude and adjusted breastfeeding rates for urban or rural residence and the associations between RF and early childhood development outcomes will be estimated with their corresponding 95% confidence intervals (95% CI). Continuous variables will be presented as mean with standard deviation (SD) or median with interquartile range (IQR). After testing for the normality of the distribution means will be compared using Student’s t test or analysis of variance. Categorical variables will be presented as counts with percentages and compared using Person’s χ^2^ test or Wilcoxon rank-sum test between groups, such as urban and rural.

For the cross-sectional analysis of the data, logistic regression and linear regression will be performed to identify barriers and facilitators of RF or infant growth and development, respectively. Interactions among the latent influencing factors will be performed using multivariable analysis to test for potential effect modification taking inequities indicators into account, such as socioeconomic status, psycho-emotional status and maternal health care access. For the longitudinal data analysis, linear mixed effect model or multilevel mixed-effects models will be performed to determine the association between RF and infant growth and development after adjusting for time dependent and static covariates. The statistical significance will be considered as a two-tailed *p* value ≤0.05. All data will be analyzed using Statistical Analysis System (SAS Institute Inc., Cary, North Carolina, USA).

### Ethical considerations

Ethical approval for this study has been granted from the Ethics Committee of Capital Medical University (No. Z2019SY001). We will follow the guidance of the Declaration of Helsinki and later amendments as well as comparable international ethical standards. All participants have signed and will always sign an online electronic informed consent before each wave of the survey during the study period.

## Discussion

In this article, we describe the protocol of an innovative responsive breastfeeding prospective longitudinal study that focuses on breastfeeding taking equity considerations into account. The study will enroll women and their singleton full term infants in five hospitals in China. As part of this study we will examine the association between RF and the growth and development of infants from birth through 12 months postpartum. We will also identify other RF barriers and facilitators from a health equity perspective. Findings will inform the policies and ways to improve the implementation of the global nurturing care framework in China.

Our study will be highly relevant for understanding how the social determinants of health interfere with breastfeeding and other aspects of responsive feeding in China. Furthermore, we will determine if and how maternal mental health affects RF and breastfeeding outcomes. This is important because lower social and economic status may affect infant feeding choices [38, 39]. Also, postpartum maternal mental health can also influence infant feeding behaviors. Indeed, maternal anxiety and stress not only increase the risk of postpartum depression, but they can also affect the quality of the interactions with their infants [40–42], which in turn can affect the behavioral and cognition development of the child [43].

Previous studies found that effective policies and concerted actions by the government, donors and civil society can increase breastfeeding rates [44]. According to the World Health Organization (WHO) guidelines published in 2017, it is necessary to strengthen social policies to support breastfeeding, such as paid maternity leave and facilitating breastfeeding in the workplace [45]. However, due to differences in culture, society, economy, and government administration, there is still a gap to achieve high standard of breastfeeding promotion across countries around the world [46].

Uneven economic development across regions in China may also affect breastfeeding outcomes [47] due to differences in access to and quality of healthcare service, i.e. health inequities. It may also be explained by the cultural diversity across regions. Hence an important strength of our study is the fact that it is multicenter and represents different regions in the country. Specifically, in more economically developed regions such as Guangzhou, and Chongqing, on the one hand, mothers are more likely to have better educational attainment and incomes, and they may have more access to breastfeeding support through the healthcare system [48]. On the other hand, given the higher maternal employment rate of women in the more urbanized area, mothers may not be in close proximity with their infants as women in less developed regions, undermining RF behaviors. Moreover, some traditional breastfeeding beliefs adversely impact exclusive breastfeeding in less developed rural areas. For instance, in Hubei Province, over half of mothers introduce complementary foods before 4 months of age because they have the traditional beliefs that breast milk is too thin to meet alone the nutritional needs of their babies, therefore, they need to add cereals or other solids for supplement before recommended [47].

Our study is highly innovative because it will examine longitudinally the association between the breastfeeding and the growth and development of infants, and as such then inform policies to fully integrate breastfeeding as part of responsive feeding. Breastfeeding is not only important as a nutrition source for babies, but also because it impacts the children’s development, including cognition and also benefits the health of the mothers [49]. To our knowledge there are no prospective studies conducted in China focusing early infant feeding and later childhood motor and cognitive development [50]. Hence, findings will be likely to help improve RF strategies in China.

According to our current research progress, we find that the the ability to recruit and follow-up partcipants varies across centers. This difference may be related to the economic, cultural, and urban-rural discrepancy and may also reflect health inequities in access to breastfeeding support. For example, the center in the large city of Chongqing has been more successful with recruitement and retention than the two rural centers in Huizhou. In order to increase participation and obtain more accurate data on breastfeeding barriers and facilitating factors, we have arrange for dedicated medical staff to supervise more clcosely the study’s staff at the centers. The PI will provide coitnuous feedback to each center based on the completeness and quality of participant’s questionnaire responses.

We acknowledge that a potential limitation of this study is that the five selected research centers do not represent all the regional variation of health care facilities across China. An additional limitation is that the CREDI has not been previously validated in China. As in any prospective study, attrition is likely to happen. In the context of China, the most likely reason is for moving to other place in search of jobs. In order to minimize attrition, we designed the study data collection points aligned with the compulsory vaccinations schedule for mothers to remember when they need to fill out their surveys and they will be rceiving reminders before their survey date. Furthermore the doctor seeing the mothers, who is a very influential figure in Chinese culture, will strongyl encourage them to fill out the survey. Lastly, if they do not complete the survey as scheduled they will be sent up to 3 reminders to do so. Despite these limitations, we believe that this study will inform evidence-based policies in China to promote breastfeeding and advocate responsive feeding from the repsonsive parenting perspective taking health equity and the social determinants of health into account. This approach is needed to further promote the healthy growth and development of Chinese infants.

## Data Availability

Data sharing is not applicable to this article as no datasets were generated for the purposes of this protocol article.

## Abbreviations

SDG: sustainable development goals
RF: responsive feeding
KAP: knowledge, attitude and practice
COVID-19: coronavirus disease 2019
CDRF: China Development Research Foundation
QR code: quick response code
CREDI: Caregiver reported early childhood development instruments
IFSQ: Infant feeding style questionnaire
EPDS: Edinburgh postnatal depression scale
ERQ: Emotion regulation questionnaire
PSI-SF: Parenting stress index-short form
PI: principal investigator
CI: confidence interval
SD: standard deviation
IQR: interquartile range
WHO: World Health Organization
